# Is ADHD a way of conceptualizing long-term emotional stress and social disadvantage?

**DOI:** 10.3389/fpubh.2022.966900

**Published:** 2022-11-03

**Authors:** Soly I. Erlandsson, Christoffer Hornborg, Emma Sorbring, Nicolas Dauman

**Affiliations:** ^1^Department of Social and Behavioral Studies, University West, Trollhättan, Sweden; ^2^Department of Sociology and Work Science, Gothenburg University, Gothenburg, Sweden; ^3^Campus Västervik, Västervik, Sweden; ^4^Department of Psychology, Université de Poitiers, Univ Rennes, Univ Angers, Univ Brest, RPPSY, Poitiers, France

**Keywords:** ADHD, overdiagnosis, attachment, family relations, social disadvantage, biopsychosocial

## Abstract

**Background:**

The prevalent, neuropsychiatric, deficit perspective on children and youth diagnosed with ADHD prohibits a multidimensional approach where socio-economic status, family stress and relationships within the families are relevant factors to examine. Assessments of ADHD through the use of rating scales and short-term interventions may lead not only to overdiagnosis but also to a reductionistic approach in the psychiatric field. This literature review aims to address research outside the prevailing discourse on ADHD as an organic brain dysfunction and broaden the perspectives on children's behavioral difficulties.

**Methods:**

The articles included in this applied, mixed-method, systematic review includes 26 peer-reviewed articles, both English and French, with a search focus on *ADHD in children and youth related to Attachment styles and relationships*.

**Results:**

In the studies reported, researchers approached correlations between ADHD and attachment in different ways, and in most cases, there was a caution to address causality. The role of parents was found to be both buffering and aggravating for the appearance of ADHD. In the French case studies, the diagnosis was conceptualized as a relational phenomenon where the child's behavior was inseparable from family member's suffering.

**Discussion:**

This review article illustrates how children's difficulties in terms of ADHD symptoms can be addressed through a paradigm where emotional and cognitive dysregulation is understood through psychosocial factors rather than as a neurological condition. In our view, to avoid an overly reductionistic and medicalized approach to children's behavioral difficulties, it is time to reiterate the value of the biopsychosocial perspective.

**Conclusion:**

Professionals and researchers need to acknowledge that becoming diagnosed with ADHD has a strong connection to economic disadvantage, social status, and familial care. The academic discourse of addressing brain dysfunctions might serve the unintended purpose of masking emotional stress and social disadvantage that manifests across generations. A biopsychosocial approach to ADHD including family, emotional history, and socio-economic issues could imply a lesser focus on medical treatment as a first choice.

## Introduction

In the relatively short history of psychiatry and mental health care, there are numerous examples of diagnoses, interventions, and explanations of human suffering that later has been questioned (1). Today, large resources are invested in research on the role of genetic and neurological factors in the etiology of Attention Deficit Hyperactivity Disorder (ADHD) [see for example (2), whereby neuropsychiatric assessment and treatment with central stimulant drugs are favored]. Faraone and Larssson's (2) view on future research advances implies a focus on unraveling the genetics of ADHD, leading to a breakthrough regarding etiology, diagnostics, and pharmacological intervention. As a consequence of the present focus on neurobiology in the discourse of ADHD symptoms, there is a risk that families seeking help for a child's behavioral problems tend to be recommended pharmacological treatment, at the expense of family therapy and individual child psychotherapy (3). The remarkably increased use of psychotropic medication can partly be explained by the fact that more and more children receive an ADHD diagnose, and some researchers have warned of a lack of knowledge about the effects on prefrontal cortex of central stimulants in children who are prescribed such medication, especially at an early age (4, 5). Consequently, it is important to address that even pre-school children (i.e., 3–5 years of age) are becoming a target of ADHD diagnostics, for example in the USA where there this group is on the rise, from 1.0 % in 2007 and 2008, to 2.4 % in 2016 (6).

It has been proven difficult to differentiate ADHD from normal variation (7). Since symptoms of the diagnosis is continuously distributed within the population – without thresholds – the prevalence is ultimately dependent on social consensus regarding the boundaries of deviation. In other words, a “natural” prevalence cannot be found out there, regardless of scientific rigor. Studies indicating that individuals with ADHD have a brain volume that is smaller compared to individuals without a diagnosis (8) has been met with criticism for shortcomings in implementation and conclusions (9). Many authors [cf. (10–12)] argue that ADHD is a heterogeneous, multifactorial problem and often with comorbidities, why the search for one explanatory factor must be abandoned. Such a view is not new, however. For more than half a century ago Anna Freud (13) shared her view on children who were perceived as having improper behavioral problems and sent to a psychiatric clinic, usually after a long series of complaints from home or from school. Freud underscored the importance of understanding the child's developmental process and suggested that the outbursts of rage and irrational behavior must be compared with the pattern of behavior within the family. Furthermore, she proposed a diagnostic procedure including a meta-psychological profile of the child, i.e., an image of dynamic, genetic, economic, structural, and adaptive data. In a recent article, Peter (14) draws our attention to the psychoanalytical view on ADHD as a complex phenomenon, requiring a multimodal approach. The author describes an uneven situation, where a well-functioning partnership between psychotherapy and medication is perceived as preferable, while critical views against the powerful, medical approach is “treated as potentially sensitive or problematic” (ibid, s. 40).

In modern times, some authors [e.g., (15–17) have advocated a biopsychosocial model in order to transcend dichotomies such as nature/nurture or individual/context, in the understanding of ADHD. While such a proposal is eligible, it is at the same time important not to throw important analytical categories – “unhelpful polarities” to quote Cooper - out with the bathwater. When it comes to psychiatric diagnoses, the gene-environment distinction is too often seemingly evaded through vague statements on “interaction.” The problem is that in the current neuro-era, any model that intertwines its components too thoroughly, risks an explanatory imbalance where “the brain” is given interpretive precedence: a BIO-psychosocial model. Cooper (16), for example, delivers a proposal for a biopsychosocial perspective by criticizing five (what we believe are partly relevant) claims that (1) there is an absence of neuro-scientific evidence for ADHD, (2) ADHD is an example of determinism, (3) ADHD rest on culturally specific judgements, (4) ADHD legitimizes the use of stimulant drugs, (5) ADHD is a medicalization of defiant behaviors. Despite his ambition of criticizing the polarity in itself, he almost exclusively criticizes one pole (e.g., constructionistic, sociological, cultural) from the position of the other pole (e.g., positivistic, materialistic, biological).

Our proposal for an application of a biopsychosocial approach rather strives to recognize the psychosocial conditions that are associated with getting an ADHD diagnosis. Such a take is needed due to the very uneven power dimensions within research paradigms and clinical discourse. A pubmed. search performed on March 3^rd^, 2022, on *ADHD and the relationship to genetics, genes or hereditary or inherited*, resulted in a total of 46.994 articles published between 1990 and 2022. Research on adverse psychosocial circumstances and the presence of high stress in families where these children grow up is sparse in comparison with the organic/synaptic discourse. This regime of biased knowledge production also manifests in clinical practice where, as Singh (15) noted already 20 years ago, methylphenidate is often being used without supporting interventions. Despite decades old evidence that early interaction patterns when infants are 6 months old is a more powerful predictor of distractibility and hyperactivity than biological or temperament factors (18), both research and clinical practice tend to be biased toward inherent organic traits rather than learned behavioral skills. Accordingly, Richard (17) discusses findings on how trauma and attachment impacts brain development and notes that psychosocial factors has been neglected in the development of ADHD symptoms: “Clinicians, when told that a child had a diagnosis of ADHD, have been found to underestimate the presence of psychosocial factors, and are less likely to ask about the possibility of neglect or abuse” (p. 483). A diversity of perspectives regarding etiology, diagnostics, and choice of therapy in children being investigated for ADHD is therefore required.

### Social disadvantage, early attachment, and emotional regulation

As Cooper (16) notes, even if ADHD is socially constructed, some persons are more likely than others to be diagnosed. An important question of course is what makes this likeliness unevenly distributed. Despite observations on how gene-environment interaction in ADHD is complex (19) and how genetic risks tend to have small effect sizes (11), there is arguably a genetic emphasis in the dominant understanding of ADHD symptoms (20). At the same time, a Swedish population-based study confirmed the link between low family income and an increased risk for psychiatric disorders, including ADHD, even after adjusting for variables concerning parental psychiatric disorders (21, 22). So, what are the implications of the neuro-discourse on how to comprehend the association between ADHD and low income? The relationship between psychiatric disorder and social disadvantage, is associated with two main hypotheses: social drift and social causation (23). The more ADHD symptoms are conceptualized as a genetic syndrome, the more the social drift hypothesis is accentuated, portraying social conditions as a dependent variable rather than a cause of symptoms, through mediating factors (24). To put it bluntly: Social disadvantage is seen as genetically inherited.

The current biological discourse may point out that individuals and families to a greater extent end up in socioeconomically unfavorable conditions due to neuropsychiatric characteristics, rather than vice versa. However, a large epidemiological study of all individuals in Sweden between 5 and 60 years old, examined the prevalence of ADHD through stratified analysis (25). While native Swedes had a higher absolute risk of ADHD than immigrants, the stratified analysis illustrated a certain complexity, where the highest prevalence was found among 10–14 years old immigrant boys from families in the middle-income group. In the light of such data, it is difficult to adhere to a social drift hypothesis and claim that children to a greater extent have an immigrant background *as a result* of neuropsychiatric characteristics. Rather, it is apparent that there is a social causation where these individuals' difficulties are a result of their history of stress being more pronounced.

Additionally, in an article on a psychoanalytical perspective on the inner world of ADHD children, Salomonsson points to epigenetics as a “third way” that can “highlight and recognize the influence of experience on a neural level without disqualifying the predictive capacity of genes” [(26), p. 1]. Considering ADHD, epigenetics may contribute to valuable knowledge about the interplay between the proposed genetic heritage and the environment, literally the impact of social context on brain development (27). Conceptually, this includes both early-life experience and how epigenetic pathways responsible for detecting the input from the environment act, and what influences phenotypic variations have across generations. Bielawski and co-authors (28) reviewed a total of 65 human preclinical and clinical studies investigating the role of epigenetic processes, the so called “protein machines” on the development of several psychiatric disorders and proposed that the alterations of epigenetic processes can have a considerable effect on mental health.

To undertake a one-sided neuropsychiatric perspective on children and youth showing behavioral problems prohibits a multidimensional, clinical approach where socioeconomic factors, family stress, and relationships within the families are relevant factors in the understanding of how symptoms develop. Research have shown that <10% in children diagnosed with ADHD has a secure attachment (29). Shifting the locus of children's difficulties from the brain to the sphere of relations is not about blaming caregivers, but rather to illuminate the inequalities in parenting, illustrated in the correlation between socioeconomic status and (some) psychiatric diagnoses (cf. Richards, 2008). Individuals from a socially disadvantaged context simply have a higher risk of experiencing stress within the family. Accordingly, Murdock and Fagundes (30) have illustrated how attachment can work as a mediator between socioeconomic disadvantage during childhood and negative health outcomes in adulthood. Wylock et al. (12) conducted a systematic literature review aimed to clarify the nature of the relationship between ADHD and child attachment and the results showed that the link still seems to remain unclear. One explanation was that many studies tend to consider ADHD as a homogeneous disorder, but the observed differences among studies could also be a matter of the methodology used to measure attachment.

Emotional self-regulation, linked to the child's attachment behavior, is mainly organized during early childhood, and developed in relationships with others, i.e., foremost caregivers (31). Difficulties with emotional dysregulation are arguably hallmark features in both attachment and ADHD discourse (32, 33), why examining correlations between attachment difficulties and ADHD-symptoms might seem irrelevant or even tautological. Yet it is important to further investigate and discuss in what way these two conditions relate to each other, since psychiatric diagnoses seldom are a result of scientific discoveries but a question of how symptoms and behaviors are interpreted and conceptualized (34). The point of departure of this review article is that the well evaluated, theoretical framework of Attachment Theory (35) could play a more prominent role, not only in the understanding of ADHD and its strong linkages to social disadvantage (36), but also in a truly biopsychosocial model of psychiatric research and clinical practice.

### Aims

An applied, mixed-method, systematic review was utilized to extend beyond the present medical ADHD discourse. Our goal was to synthesize quantitative and qualitative research focusing on early relations/attachment in association with children diagnosed with ADHD. In consideration with an ethical and empathic attitude to children's behavioral problems the prevailing biomedical view demands to be interrogated and replaced with a biopsychosocial disease model. The main goal of this article was:

To investigate the relationship between ADHD and attachment. Secondly, to discuss the results in relation to how psychosocial factors are required in the understanding of ADHD symptoms. In line with this goal our research question was: *How does symptoms of ADHD relate to psychosocial conditions such as attachment, emotional growth, and family stress?*

## Method and procedure

For guidance in performing this literature review we used the J. Briggs Institute's Methodology for Mixed Methods Systematic Reviews (MMSR), (37), based on two research paradigms, i.e., positivism and constructivism, of which we followed the latter paradigm. According to the authors, MMSRs has become an important method in health care research but is still under development. The mixed method can bring together quantitative and qualitative evidence “to create a breadth and depth of understanding that can confirm or dispute evidence and ultimately answer the review question posed” (ibid). Hong and colleagues (38) identified two types of frameworks that were dominant in mixed method reviews, referred to as the convergent and the sequential approach. We have adopted a convergent, integrated approach, which allows for a combination of quantitative and qualitative data. Allowing for an inductive and a deductive approach as in the mixed-method systematic review has the advantage of providing a well-founded view of the research subject. Terminology used throughout the text is clarified as Key terms (see Table 1).

**Table 1:** Key terms.

**Attachment theory**	A psychological theory stating that young children are genetically programmed to develop a strong relationship with at least one primary caregiver. According to the theory, the quality of this relationship may impact the child's physical, psychological, and developmental well-being.
**Biopsychosocial model**	The notion that a person's medical status should not solely be understood through biological factors, but also through psychological factors such as emotional stress, beliefs and coping methods, as well as social factors such as economic situation, relationships and social status.
**Neo-Kraepelinian**	A revival of the biomedical discourse of Emil Kraepelin, where psychiatric symptoms are primarily seen as a malfunction in the brain, rather than a result of psychological forces or social factors.
**Neuropsychiatric**	A medical field that conceptualises cognitive and behavioral symptoms as a result of how neurobiological traits provide individuals with different resources in tackling external demands.
**Psychodynamic**	An approach to psychology and psychiatry that emphasizes the impact that unconscious thoughts, emotions, and impulses have on human behavior and health, and especially how these relate to early experiences and learning.
**Social drift hypothesis**	The notion that psychiatric symptoms increase the risk of drifting into a lower social class and decreases the chances for upward social mobility.
**Social causation hypothesis**	The notion that economic disadvantage increases the risk of experiencing psychiatric symptoms.

### Search history and eligibility criteria

For the search of articles according to the research question, the following databases was used: Psychlitt., Psychinfo, and Cairn. The searches were performed on March 24^th^ (APA Psychinfo), April 1^st^ (Psychlitt.), April 21^st^ (Cairn) and May 4^th^ (Psychlitt. and Psychinfo). Thesis, short, descriptive studies, and articles in other languages than English and French were initially removed from the records. Criteria for inclusion were empirical studies, systematic review studies published in peer-reviewed journals and studies that were eligible for ADHD in children and youth. The results of the searches were organized and specified as the following profile:

Peer-reviewed articles (English-spoken) with the search terms: *ADHD or attention deficit hyperactivity disorder, Children or kids or youth or child, Attachment styles and relationships, or attachment styles in child development*. Peer-reviewed articles (French-spoken) with the search terms: *hyperactivité (hyperactivity), TDAH (ADHD), enfant (children), adolescent, famille (family)*.

**Figure 1:**
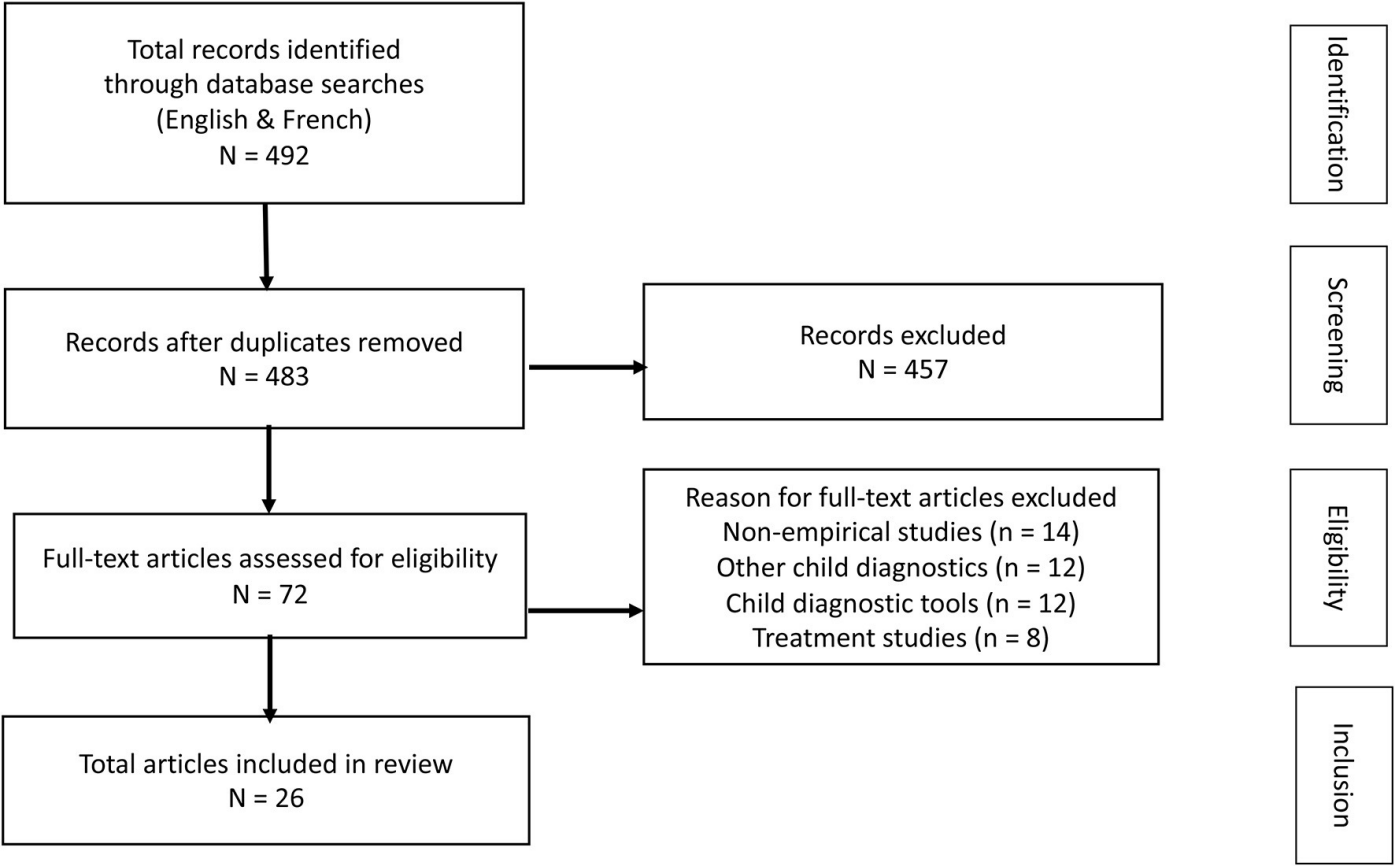
PRISMA Flow Diagram used in the review is adapted from Moher et al. (39).

The total records identified after the initial database searches were 492. Removal of duplicates left 483 articles. The vast majority of articles written in French referred to a biomedical or neurodevelopmental perspective on ADHD. Articles that did not focus on psychosocial factors associated with child and family, such as opinion and theoretical articles, were excluded. Furthermore, some articles were excluded due to irrelevant content, i.e., focusing on other diagnoses or on treatment evaluation. Full-text articles assessed for eligibility amounted to 72 articles. In order to decide on eligibility the authors examined the full-text articles (*n* = 72). In total, 26 articles were found eligible for being included. The procedural steps taken throughout the review process are shown in Figure 1.

### Quality appraisal and synthesis of data

Throughout a narrow reading of included articles, the authors made their quality assessments after careful considerations and agreements improved by the research team. This implied a main focus on methodological rigor, i.e., if research questions were clearly formulated and could be answered with the data presented. Articles that did not meet with these criteria were sorted out. In the next step, the main findings of each of the articles were summarized and displayed in Table 2, where authors, research methodology and a brief summary of the content appear. The distribution of methodology of studies included were: Retrospective/Case control studies (9) – Case/multiple case studies (6) – Correlational studies (6) – Review studies (5).

**Table 2:** Articles included in the review analysis (*n* = 26).

**Reference**	**Study design**	**Participant/method**	**Summary results**
Al-Yagon et al. (40)	Case control study	100 children, age 11–12, 50 with ADHD, 50 with TD	Children with ADHD reported higher incidents of insecure attachment to the father, and lower trust and closeness to the mother, in comparison to children with TD (Typical development).
Al-Yagon (41)	Case control study	100 children, age 11–12, 50 with ADHD, 50 with TD	The child-mother attachment affected all measures for EF (executive functions) while the child-father attachment did not affect these measures.
Bourrat (42)^*^	Case study	A boy's follow-up from 6 to 24 months, with his mother	Psychotherapy unraveled a traumatic experience in the mother's own childhood (death of her younger brother) that resonated with her helplessness in front of her second child's hyperactivity (“le petit frère”).
Clarke et al. (32)	Case control study	38 boys, age 5–10, 19 with ADHD, 19 with TD	The ADHD group received lower scores on all tests associated with attachment (Separation Anxiety, Self- Interview, Family Drawing).
Dalaire and Lafortune (43)^*^	Review study	23 studies (family factors) based on DSM diagnosis	Additional burdens in parents of children with ADHD (alcoholism, drug dependency, anti-social personality, mood disorder, divorce, low income) are associated with coercive discipline and less efficient coping strategies in family conflicts.
Duc Marwood (44)^*^	Case study	A 6-year-old boy diagnosed with ADHD	Tales in psychotherapy helped the child identify with the characters and their emotions (fear, courage, anger), which unraveled conflictual relationships within the family that was behind hyperactive and inattentive conducts (father's abuse and domestic violence).
Edel et al. (45)	Retrospective case control	73 adults with ADHD	Adults who subjectively remembered their own mother's ADHD symptoms from growing up, had to a greater extent attachment problems and difficulties with emotion regulation in the present.
Eyuboglu and Eyuboglu (46)	Case control study	48 newly diagnosed untreated adolescents with ADHD, 51 in a control group	Adolescents with ADHD had a higher degree of avoidant attachment. Affiliation scores correlated with ADHD severity.
Finzi-Dottan et al. (47)	Correlational study	65 children with ADHD, age 7–15	The child's temperament in combination with different parenting styles gave rise to different attachment styles in the child. Some parenting styles parenting style can aggravate the child's difficulties with self-regulation, and lead to insecure attachment.
Guinard (48)^*^	Multiple case study	Clinical assessment with drawings and projective tests (36 childrens, aged 6–11, 31 boys).	Exclusive attention from the mother, rigid control over the clinician's presence and explosive conducts characterizes children who turn out to be highly sensitive to the evocation of family conflicts.
Hornstra et al. (49)	Case control study	Children age 8–12, 45 with ADHD, 57 with TD	There were no significant differences between the ADHD group and the TD group regarding attachment or trust in parents. Attachment might be linked to ODD (oppositional defiant disorder) and CD (conduct disorder) symptoms.
Hurt et al. (50)	Correlational study	110 children with ADHD, 108 mothers, 53 fathers	Higher warmth from fathers was associated with higher peer acceptance, and higher exercise of power from the father was related to lower peer acceptance, but both correlations applied only at low levels of family loneliness. The mother's warmth and exercise of power were not related to either.
Latimer et al. (51)	Review study	47 studies on DBD (umbrella concept for ADHD, ODD and CD.	Risk factors for DBDs (Disruptive Behavior Disorders) were prenatal smoking and alcohol use, prenatal viral disease, stress and anxiety in the mother, low birth weight, complications associated with birth, parental stress and parental style during infancy, early neglect, adoption, and separation. In the field, there has been a disproportionate focus on certain risks at the expense of others.
Lemelin et al. (52)^*^	Review study	Number of studies not indicated (focus on parenting and family interactions, from 1985 to 2005)	Additional burdens in parents of children with ADHD (marital conflicts, psychopathology, drug abuse, coercive discipline, low self-esteem) threatens the consistency and attentiveness of their attitude toward their children.
Maguire et al. (53)	Review study	30 studies on school-aged children (of which 4 focused on ADHD)	Children with difficulties at school, ADHD symptoms or deviant behavior, are overrepresented in having a background of neglect or emotional abuse.
Metz and Thévenot (54)^*^	Multiple case study	Projective tests of 17 children diagnosed with ADHD, 14 boys, age 8–12. Focus on two cases: a boy of 8 and a girl of 9, and their parents	Psychotherapy helped parents to identify the power in the relationship to their child with ADHD, as they struggled to distant themselves from their own familial history of trauma (violence, abuse, bereavement of children in their own parents' life).
Nahas et al. (55)^*^	Correlational study	110 children with ADHD	The parent's attachment style and educational technique was a risk factor for the development (or worsening) of ADHD-like symptoms. The less parental involvement in the child-parent interaction, the more hyperactivity in the child.
Petot (56)^*^	Case study	A 6-year-old boy diagnosed with ADHD, met again at 13	The child's hyperactivity functioned as a defense against anxiety separation and led to mutual hold with his mother through constant attention to him; oppositional defiant behavior resulted from the escalating of mutual frustration between the boy and the adults over the years (at schools and family).
Rasmussen et al. (57)	Correlational study	67 children, ages 7–12, and their mothers	ADHD symptoms in the mother and high RSA (Resilience in Adults) in the mother correlated with a positive treatment outcome for the child. There was no correlation between the mother's attachment style and treatment outcome for the child.
Rasmussen et al. (58)	Correlational study	64 mothers of children with ADHD	Self-reported ECR (insecure attachment style) correlated negatively with self-reported RSA (Resilience in adults). Self-reported ADHD correlated with self-reported RSA. A secure attachment in the mother may contribute to the development of maternal resilience, which may in turn be an important factor in parenting.
Scharf et al. (59)	Correlational study	508 adolescents in 'junior high'	Adolescents with an insecure-ambivalent attachment had a higher degree of ADHD. Insecure-ambivalent and insecure-avoidant attachment correlated to the same extent with social adjustment difficulties.
Sempio et al. (60)	Case control study	72 children: 36, age 4–5, of which 24 in the risk zone for ADHD. 36, age 7, of which 24 in the risk zone for ADHD	Children with a higher degree of ADHD received lower scores on attachment security, based on F-SAT (Family Separation Anxiety) and S-SAT (School Separation Anxiety). Attachment deficit may be an underestimated factor in the diagnosis of ADHD.
Sochos and Yahya (61)	Case control study	98 parents of children and adolescents with ADHD, 153 parents in the control group	Parents of children with ADHD had more difficulties in their partner relationships, but the parental groups differed only when attachment style was controlled for. Parents of children with less severe ADHD were more likely to experience relationship problems.
Sourgen (62)^*^	Case study	An 8-year-old boy, with his mother	Psychodynamic psychotherapy (drawings and free associations) helped the mother and her child in dealing with the anxiety separation that sustained the mutual hold behind inattentive and hyperactivity conducts.
Storeboe et al. (63)	Review study	29 studies on the association between attachment and ADHD	There is a clear association between ADHD and insecure attachment. Parental involvement in combination with environmental factors was associated with ADHD in children. Adults with ADHD have to a greater extent a more insecure attachment style.
Özyurt et al. (64)	Case control study	61 children, ages 8–12 with ADHD. 87 children in the control group.	Children with ADHD had more difficulties with emotion regulation and empathic ability. Mothers of children with ADHD had a higher degree of insecure attachment and more difficulties with emotion regulation (DERS).

ADHD, Attachment and the child-parent relationship.

* Indicates French article.

Whether qualitative or quantitative, all eligible articles were converted into a text. To reach an approved level of structure and synthesis of the reviewed articles a comprehensive, close reading of each part were made by two authors (SIE, CH). The next step implied to characterize the content by searching for common denominators, i.e., themes describing those denominators, a process undertaken by the first author (SIE). Searching for themes was guided by Braun and Clarke (65) who declared that “a theme capturers something important about the data in relation to the research question.” Furthermore, a theme should represent a level of “patterned response or meaning within the data set” (ibid, p.82). In the final stage of the outline of the results, the themes were approved by all other participating authors. In order to give the reader's access to the French-spoken literature included in the review, abstracts of the French articles were translated into English by one author (ND), see Supplementary material including abstracts of 8 such articles.

## Results

The main focus of the results in the review is how ADHD relate to attachment, including the family environment. Included articles had varying research methods and focuses (see Table 2), and were themed (65) with the following headings and sub-headings:

**• ADHD, attachment, and the family environment**
*Competing discourses about the interaction between ADHD and attachment*
*ADHD, early development, and interactions with environmental stimuli*
*Narratives of family bonds in parents of children with ADHD*

### ADHD, attachment, and the family environment

In focus of this review, attachment difficulties were associated with ADHD symptoms in a different number of ways (63). Children diagnosed with ADHD had lower scores on tests associated with secure attachment (32), reported higher incidents of insecure attachment to the father, and lower trust and closeness to the mother (40). These attachment patterns were transferred to other contexts such as the school environment, where ADHD symptoms correlated with separation anxiety (60). However, Hornstra and colleagues (49), in a study of children between 8–12 years of age, found no connection between attachment and ADHD, but on the other hand that attachment can be an important factor for the development of ODD (Oppositional Defiant Disorder) and CD (Conduct Disorder). One systematic review of children with difficulties in school and behavioral problems (among them ADHD symptoms), showed that these children more often have a background of relational trauma, such as neglect or emotional abuse (53).

**Competing discourses about the interaction between ADHD and attachment**

In the studies found in this review, the researchers approached correlations between ADHD and attachment in different ways, and in most cases, there was a caution to address causality. However, differences in how results were interpreted and formulated could indicate disparate basic assumptions among researchers. Scharf and coworkers (59) thus found that adolescents with an insecure-ambivalent attachment had a higher degree of ADHD symptoms, suggesting that relational patterns can be a *developmental precursor* for ADHD and adjustment problems in school. Eyuboglu and Eyuboglu (46) on the other hand found that avoidant attachment issues among adolescents correlated with ADHD severity but framed this as an indication of how ADHD *affects other aspects of life*. In a similar way, several studies investigated the relationship between child and parent characteristics but emphasized different aspects when it came to the direction of influence. Özyurt and coworkers (64) found that mothers of children diagnosed with ADHD had a higher degree of insecure attachment and suggested that the emotion regulation skills of the mother are vital for the child's development process. Likewise, Nahas and fellow researchers (55) stated that both parent attachment style and parental educational techniques were risk factors for the development or worsening of ADHD symptoms. In that study, less parental involvement and supervision was associated with a heightened extent of hyperactivity in the child.
In a review by Weissenberger and colleagues (66) parenting style was found to be both buffering and aggravating for the appearance of ADHD. Parenting style including warmth, communication, and clear boundaries for the child (authoritative parenting) was associated with better educational outcome, less symptom severity and lower rates of drug abuse and addiction for the child diagnosed with ADHD. Parenting including harshness, neglect, and strict parenting behaviors (authoritarian parenting) was associated with exacerbated symptoms with inferior academic performance and an increased risk of being diagnosed with CD. Other researchers approached correlations between parental behavior and ADHD in an opposite way. Finzi-Dottan and colleagues (47) thus argued that temperament factors among children with ADHD evoke certain reactions among parents, which aggravate the child's regulation difficulties and lead to an insecure attachment. Another study found that parents of children with ADHD had more difficulties in their partner relationships, especially if they also had high attachment avoidance, making parents more unable to enjoy satisfaction in adult relationships (61). These kind of studies focuses on parent stress due to managing the child's behavior rather than how family aspects contribute to ADHD symptomatology in the child (67). Such a discourse is in line with older observational studies, which have shown that mothers of hyperactive children initiate less interaction, and are more critical and disapproving toward the child, suggesting that hyperactivity evoke negative behaviors from the mother (68). One of the studies in this review found that ADHD adults who from their own up-bringing subjectively remember ADHD symptoms in their mother, are at a greater risk of experiencing difficulties with emotion regulation in the present (45). Attachment difficulties among individuals with ADHD are thus seen as an effect of (undiagnosed) ADHD among primary caregivers since this had implications for parental style during childhood.
In summary, the results illustrated competing discourses about the interaction between ADHD and attachment. Correlations between parental behavior and difficulties expressed from a child could mean either that the former contributes to the latter, or the other way around, i.e., that inherent dysfunction in the brain of the child affects the interaction and the behavior of caregivers. Clarke and coworkers (32) point out that research on ADHD tends to focus on the latter, where psychosocial contributors are regarded as peripheral to the development of the disability. In the review of Storebø and coworkers, the relationship between attachment security and ADHD was described as follows: “ADHD and insecure attachment … are mutual risk factors; when one of the conditions occurs, there is an increased risk for developing the other” [(63), p. 193]. Thus, while the results mostly support a tangible connection between insecure attachment and ADHD symptoms, there are differing interpretations when it comes to the direction of influence between the characteristics of the child and the behavior of the caregivers.

**ADHD, early development, and interactions with environmental stimuli**

Lemelin and coworkers (52) found that marital conflicts, psychopathology, drug abuse, coercive discipline, and low self-esteem in parents of children with ADHD seriously impacted their relationship with the child. Dalaire and Lafortune (43) reported similar behavioral strain (alcoholism, drugs dependency, and mood disorder) in parents of ADHD children, associated with more coercive discipline on their children, and less efficient coping strategies in dealing with family conflicts. While most of the literature on ADHD and attachment focuses on emotional development, Al-Yagon with colleagues (40, 41) explored how attachment relates to executive functions, i.e., cognitive skills associated with goal-directed behavior. According to some research, the development of these higher cortical functions requires interactions with environmental stimuli (69). In one of the studies, impairments in executive functioning among the children was affected by the relationship with the mother (41).
Other results showed that high resilience in the mother was associated with a positive treatment outcome in the child (57). In one study, self-reported maternal resilience significantly correlated with attachment style, suggesting that attachment security may contribute to the perception of resilience, which may in turn be an important factor for children's developmental outcome (58). Rasmussen and colleagues also found that maternal resilience correlated with ADHD symptoms in the mother, and that these symptoms was associated with a positive treatment outcome in the child. This might be due to the fact that parent and child ADHD similarity is associated with the parent being more empathic with the child's difficulties (70).
Even when ADHD is mostly considered a neurological disorder, there seem to be several environmental factors affecting the condition. One study found that parent style from the father was associated with peer functioning of boys diagnosed with ADHD, but only among boys that experienced low levels of family loneliness (50). As Sempio and coworkers [39, p. 70–71] write: “Factors such as temperament and arousal … are not, on their own, powerful predictors of an insecurity pattern of behavior … they seem to have a significant impact only when other risk factors, such as poor parenting, economic hardship or difficulties of attachment are also present.” In a study of risk factors for Disruptive Behavior Disorders (which at the time of the study included both ADHD, ODD, and CD), Latimer and coworkers (51) found links to prenatal smoking and alcohol use, prenatal viral disease, stress and anxiety in the mother, low birth weight, complications associated with birth, parental stress and parental style during infancy, early neglect, adoption, and separation. The authors conclude that in this area of research, there has been a disproportionate focus on certain risks at the expense of others.
In summary, several studies confirmed the relationship between adverse psychosocial circumstances and ADHD symptoms. Results suggest that some factors only have a significant impact in combination with certain other factors. Also, instead of only investigating the correlation or the direction of influence between attachment and ADHD, attachment can pose as an independent variable that contributes to resilience, having a moderating impact on the genesis of symptoms.

**Narratives of family bonds in parents of children with ADHD**

Among the eight studies included in this review, some commonalities can be identified in the family dynamics within the French community. The authors describe a *mutual and paradoxical hold* between the child and the parents. The dynamics of ADHD is presented in the following way by Petot [60, p. 97]: “the more the child is inattentive, lacking in foresight and impulsive, the more his parents must be watchful, provident and vigilant *instead of him*” (original italics). Having to pay continuous attention, the parents are deprived of their own thoughts and feelings in their relationship to the child, and can hardly be involved personally in the interaction, as they most of the time only react to his lack of self-regulation. Petot (56) reported the case of a 6-year-old-boy diagnosed with ADHD, who subsequently developed ODD. The family bonds associated with ADHD was defined as a self-perpetuating process, in which the child's inattentiveness and the parents' required control over him resulted in escalating frustration on both sides, with subsequent punishments and provocations. As a consequence of this self-perpetuating process, Metz and Thévenot (54) suggest that parents feel discouraged to interact with their child, who repeatedly ignore their words and their own feelings. Moreover, the parents see the child as a stranger in the family, who is driven out of control by the disorder. Unwittingly, and instead of psychotherapy, they adopt a discourse that reify their child as having a biological disorder. Petot (56) and Sourgen (62) identified anxiety separation as a core threat to which both parents and their child defend themselves against, unconsciously turning it into turmoil, worries and frustration, which could lead to a highly seductive attitude. Accordingly, Guinard (48) reported examples of relational demands from children with ADHD, requiring an immediate attention drawn back to them when other interactions were initiated.
Families encountered in consultation for ADHD were afflicted by traumatic events in the family history, where the parents' helplessness in front of their child's behavior resonated with past circumstances they were not able to make sense of. Bourrat (42) reported the case of a 6 months old boy, whose mother felt overwhelmed by guilt for not being able to temper and interact with him (her second child). The childbirth had been painful, and the boy had to be hospitalized, after which both parents worried for the unrest and inattentiveness in the little boy. In her own childhood, the mother witnessed the death of her younger brother who died in a traffic accident. The psychotherapy process helped the mother identify her fears for her second child, that resonated unconsciously with the horror of her younger brother's death and interactions with the boy was restored. As suggested by Metz and Thévenot (54), parents who from their own family history face struggles without closure, may feel resourceless when it comes to raising a child diagnosed with ADHD. In psychotherapy, parents could process unconscious emotions that interfered with contemporary relations between parents and children. For instance, a father could express his reluctance to exert authority with his child, remembering how violent his own father was. The authors suggest that these kind of events have a powerful impact on the parents' relationship to their child. Duc Marwood (44) suggested that inattentiveness and disruptive behaviors sometimes can function as an unconscious strategy to avoid thoughts and feelings connected to violence witnessed in the family. In line with Metz and Thévenot (54) the author considered that, in some instances, hyperactivity should draw the attention of health professionals to emotional suffering that the child is unable to contain and speak about.
Furthermore, the authors suggest that family members, and especially the child with ADHD, are *highly sensitive to words* that may evoke inner conflicts. Sourgen (62) and Duc Marwood (44) describe boys who display strong reluctance in talking about themselves. Metz and Thévenot (54) met parents who were distant from feelings and thoughts about their own relationships, apart from the frustration their child with ADHD evoked in them. The children didn't want to talk or listen to the clinicians, and behaved in an aggressive manner, claiming that they could not narrate their feelings. Similarly, Guinard (48) observed that a majority of the children she met were reluctant to share their imagination or play with their inner fantasies when being shown pictures they were expected to comment on (like the Rorschach Inkblot test). Instead, the children wanted to control the activities and give order to the clinician, as if they were authoritative adults. For instance, one daughter would immediately ask the clinician to mind her business, requiring her mother to shut up, when the latter recalled a period of hospitalization during which the daughter and the mother were separated from one another. In doing so, the child seemed to defend herself from painful emotions associated with the separation.
In summary, the psychodynamic case studies illustrated how the “ADHD behaviors” of the child were inseparable from ongoing relational processes within the family. Both children's and parent's behavior sometimes functioned as defenses from unpleasant emotions, and these were sometimes connected to earlier events in the family history. Through psychotherapy, both children and parents could receive help in becoming aware of and processing emotions (i.e., learning to self-regulate, instead of acting out dysfunctional behaviors as an automated avoidance strategy).

## Discussion

There is a noticeable risk of over-diagnosis when the treatment paradigm primarily targets the child's individual traits and overlooks contextual factors such as economic hardships and complications in family life. This review article has given examples of how the later are important variables in the understanding of ADHD symptoms. Several of the case studies based on psychotherapy explore the relationships between the parents' stress and their children's behavior (43, 52, 54, 61). Dalaire and Lafortune describe the presence of severe distress in families of children with ADHD, such as divorce, drug dependency and economic disadvantage. Early relationships also seem to have implications for how some cognitive skills develop in children with ADHD (41). Important to note is that while several studies in this review identified correlations between attachment deficits and ADHD, Hornstra and colleagues (49), found no such connection, but on the other hand that attachment related to ODD and CD. In the light of these findings, one might ask whether some behavioral difficulties are more connected to attachment and environmental conditions than others. Or if the findings rather illustrate the complexity of environment-behavior relations, and the fallacy of sorting children's adjustment difficulties into medical boxes.

Being recognized as someone who have ADHD is not seldom felt as a relief and a sense of social belonging (71, 72). The experience of “belonging” underlines the importance of a socially inclusive treatment of children who risk ending up outside normality. Contrary, there is a risk that children with deviant behavior might become recipients of an individualized discourse where the focus lies on vague labels rather than on the lifeworld of the person. As an example, a child with behavioral misconducts was helped in psychotherapy to express strong emotions being tied to hyperactivity and inattentiveness associated to abuse and domestic violence (44). According to such a treatment paradigm, a child's inability to conform to the prevailing pattern of behavior might be a way of expressing an important message, which can contribute to recognizing unhealthy and pathogenic modes in society, school, or family (13). We argue that the mainstream discourse on ADHD, despite scholarly ambitions of describing a “multifactorial” condition, tends to shift the focus from a relational perspective on the subject, to a focus on the object, where phenomena such as attention, impulsivity, and executive functioning, are regarded as static traits of the brain, rather than a reflection of stress, social disadvantage, or lack of learned skills.

To avoid an overly reductionistic approach to children with behavioral problems, it is high time to reiterate the value of a biopsychosocial perspective, that gives equal attention to all its components. In a published essay in Lancet Psychiatry, Gask (73) describes the emergence and dismantling of the biopsychosocial model and highlights the importance of time, i.e., why the patient is applying for professional help at a specific moment in life. She emphasizes the importance of recording how the biological, psychological, and social factors vary, not only between disease episodes but also over time and circumstances in the patient's life. This is a highly important aspect in the understanding of ADHD symptoms, especially in an era where young people who have been diagnosed in childhood are beginning to request their “neuro-disorder” to be reconsidered [for a Swedish context, see (74, 75)]. Previous research on health in adults has shown how attachment can be highly relevant for a biopsychosocial model, since it can work as a mediator between “the social” (low SES) and “the bio” (symptoms) (30). Accordingly, we argue that the relational perspective inherent in the attachment paradigm can contribute to a holistic understanding of children's emotional and behavioral difficulties by addressing how external circumstances can have impact on the development of the child's “internal working models, *which reflect the outer lived experiences on an inner level*” [(63), p. 187]. When it comes to ADHD, the academic discourse of overemphasizing the brain, might in fact serve the unintended purpose of masking the emotional stress and social disadvantage that manifests across generations.

As Latimer and coworkers (51) concludes, in the research area of disruptive behavior disorders, which at the time of their study included ADHD, there has been a disproportionate focus on certain risks at the expense of others. Our main argument here is that academic discourse is not solely based on scientific facts, but on how researchers across time and space choose to focus on different empirical arenas and theoretical models, due to different core assumptions. The difference between developmental paradigms such as attachment or psychodynamic theory on the one hand, and the neuropsychiatric discourse on the other, is that the former to a *greater* extent emphasizes how psychological characteristics (e.g., impulse control, executive functions, ability to concentrate, relationships) develop in harmony with the social environment. At the same time, it is important not to land in a simplified either/or approach to “organic” syndromes such as ADHD vs. “learned” behaviors such as attachment difficulties since research illustrates how these (along with sociological variables) are tightly interwoven (63). Despite this, we argue that the view on the relationship between neuropsychiatric traits and environmental factors needs to be much more nuanced. To illustrate, it would most certainly be far more acceptable in contemporary academic discourse to claim that attachment difficulties could *accentuate* ADHD symptoms, than to claim that it actually *creates* them. Such a discourse contains a puzzling view on the link between brain and environment. Since ADHD is continuously distributed in the population, where getting a diagnosis is the same as passing the clinical threshold or cut-off, *any accentuation of ADHD traits must be equivalent to etiological cause*.

### Strengths and limitations

The present review article demonstrates how complicated the phenomenon of ADHD is. We believe that it is both an opportunity and a duty as researchers to present the breadth of knowledge that different scientific disciplines can offer. One of the strengths of this contribution is that the everyday life of those most affected by ADHD diagnoses have been given attention, which may offer a more integrated and broad view on the research area. Additionally, alternative explanatory models for children's inattention and hyperactivity and the importance of family relationships were invoked in the French studies. While all authors have a research affiliation within behavioral and social science and three authors are experienced in clinical psychology, there is a width among the researchers' scientific backgrounds including sociology, medical anthropology, psychoanalysis, and the interdisciplinary field of child and youth studies. Some limitations of the study must also be disclosed. Many of the studies included in this review have small samples and the limited number of reviewed articles may jeopardize the conclusions that can be drawn. Furthermore, leaning on a review method like the mixed-method systematic review implies a number of procedural steps, of which we have been able to follow some, and not others. We have, however clarified in detail how the review process was carried out.

### Implications for future research

No doubt, the vast majority of research on ADHD continues to rest on neurological assumptions about children's attributes as a unified and inherent, biological body. Given the unequal allocation of resources available in contemporary ADHD research, there is reason to consider the long-term consequences that the lack of a biopsychosocial research paradigm has for scientific discourse and reliability. But also, what the effect is for those youngsters who are expected to live their lives with a label that implies that to fit into society they need to be dependent on drugs. Future research and clinical practice can benefit from more openness and less rigid beliefs in the understanding of behavioral difficulties in children (and adults). Such a paradigm shift includes more resources for the humanities and social sciences in studies of hyperactivity, concentration and emotional regulation. Otherwise, there is a risk of another 40 years with a continuing, rising curve of even younger children who are medicalized and defined as having a disease/disorder. To perceive children as subjects whose development is dependent on a secure and trustful social context can raise parents' (and society's) sensitivity to children's needs of sharing their feelings of frustration. The child psychiatrist Sami Timimi has offered a treatment option “The Rational Awareness Program” for parents and their children struggling with challenging behavior (76). This family therapy prioritizes building relationships, over controlling the behaviors and symptoms of the child. Instead of medicalizing the child's behavior through a diagnosis of the brain, focus is on giving support and help to the parents, which serves as a treatment also for the child. In accordance with this view, psychotherapeutic techniques (26, 77–79) could be alternatives to the current, often first-of-choice, central-stimulant medication. Within a psychotherapeutic framework, children diagnosed with ADHD can receive unique attention allowing them to communicate what they perceive as most disturbing in their life.

## Conclusions

This review article has investigated and discussed how children's behavioral difficulties labeled as ADHD can be understood through factors such as attachment, family relations and emotional history (and indirectly also broader sociological variables). Such a focus does not deny that individuals have differing genetic vulnerabilities for developing psychiatric symptoms, but rather strives to re-establish a biopsychosocial model that has become vaguer in a neo-Kraepelinian era. Even though Anna Freud (13) wrote her book more than half a century ago, her way of comprehending a child with severe behavioral problems is remarkably inclusive when it comes to etiological considerations, and it has none of the reductionistic ambitions guiding much of the biomedical theorizing that dominates the view on ADHD today. Such a position opposes the prevailing biomedical view, not because it denies that we are biological creatures, but because it refuses to reduce behavioral issues into a biological disturbance and advocates a model that puts equal attention on biological, psychological, and social factors in understanding and treating ADHD symptoms.

## Author contributions

SE wrote the first draft and was responsible for guidance in the process of writing the paper. The search for publications was done by SE, CH, and ND. All authors participated in reading and summarizing the review articles and writing the result section. All authors contributed to manuscript revision, read, and approved the submitted version.

## Conflict of interest

The authors declare that the research was conducted in the absence of any commercial or financial relationships that could be construed as a potential conflict of interest.

## Publisher's note

All claims expressed in this article are solely those of the authors and do not necessarily represent those of their affiliated organizations, or those of the publisher, the editors and the reviewers. Any product that may be evaluated in this article, or claim that may be made by its manufacturer, is not guaranteed or endorsed by the publisher.
