# Erratum: Is ADHD a way of conceptualizing long-term emotional stress and social disadvantage?

**DOI:** 10.3389/fpubh.2024.1491023

**Published:** 2024-09-17

**Authors:** Frontiers Production Office

**Affiliations:** Frontiers Media SA, Lausanne, Switzerland

**Keywords:** ADHD, overdiagnosis, attachment, family relations, social disadvantage, biopsychosocial

Due to a production error, “Peter (2021)” was not cited in the article. The citation has now been inserted in the section **Introduction**, Paragraph two, and should read:

“In a recent article, Peter (14) draws our attention to the psychoanalytical view on ADHD as a complex phenomenon, requiring a multimodal approach. The author describes an uneven situation, where a well-functioning partnership between psychotherapy and medication is perceived as preferable, while critical views against the powerful, medical approach is “treated as potentially sensitive or problematic” (ibid, s. 40).”

The publisher apologizes for this mistake. The original version of this article has been updated.

## References

[1] Peter S. An (un)happy marriage: chilad psychology with children who are medicated for ADHD. J Child Psychother. (2021) 47:32–53. 10.1080/0075417X.2021.1945657

