# Supplementary material for: Is ADHD a way of conceptualizing long-term emotional stress and social disadvantage?
Source: Front Public Health. 2022 Nov 3;10:966900. doi: 10.3389/fpubh.2022.966900 (PMC9680974; doi:10.3389/fpubh.2022.966900)
Supplement: Supplementary file 1 [file Table_1.docx]

**Appendix.**

Eight abstracts of French articles (English translation) included in Table 2: ADHD: Attachment and the child-parent relationship.

| **Dalaire & Lafortune. (2008). Trouble de déficit de l’attention: facteurs génétiques et familiaux. Une recension des écrits.**  ‘’*Attention deficit disorder: genetic and familial factors. An inventory of writings on the subject.’’*  Keywords: ADHD, Genetics, Family environment. | Attention deficit disorder with or without hyperactivity (ADD/ADHD) is one of the most common disorders in children. The diagnosis is associated with several comorbidities, including troubles of opposi- tion/defiance, anxiety, depression and learning difficulties. The complexity of the syndrome has led researchers to try to understand its genetic and environmental origins. Twin studies and family studies of adoption show that the genetic heredity of ADD/ADHD is approximately 0.75. Three main genes seem to be involved in the development of the disorder : The 5-HTT, the DRD4 and the DAT1. The family environment also seems to play a role in the development of ADD/ADHD. Psychopathologies in the parents, their personality traits, as well as parenting techniques seem to be different with children suffering from an ADD/ADHD diagnosis, and related to the symptoms associated with the ADD/ADHD. |
| --- | --- |
| **Lemelin et al. (2006). Les caractéristiques familiales des enfants présentant un trouble déficitaire de l’attention avec ou sans hyperactivité: recension critique des écrits.**  ‘‘*Characteristics of families with children diagnosed with ADHD–a critical literature review.*’’    Keywords: Attention Deficit Disorder, Attention Deficit Disorder with Hyperactivity, Family, Parental Characteristics, Childhood (birth-12 yrs) | This article provides a critical summary of the most recent research on familial and parental characteristics of children with attention deficit disorder with or without hyperactivity (ADD/H). Most of the studies on families with children who have ADD/H suggest that the presence of ADD/H tends to be linked to a less functional family, especially if the child has concomitant CD/ODD. In addition, the psychopathological and non-pathological characteristics of the parents (attributing causality, feeling competent, personality, marital conflicts) are linked to the manifestation of symptoms in the child. The development of an ADD/H symptomatology is therefore influenced by family and parental characteristics, especially if the child exhibits concomitant CD/ODD. Most of the articles found on this subject seem to indicate that there is a reciprocal influence among the characteristics of the family, the parents, and the CD/ODD symptoms. In this article, we present a critique of the existing research and make suggestions regarding future research. |
| **Petot. (2004). Pourquoi l’hyperactivité infantile évolue t-elle fréquemment vers le ”trouble oppositionnel avec provocation”?**  *‘’Why does attention-deficit/hyperactivity disorder often turn into oppositional defiant disorder?’’*  Keywords: Attention-deficit/hyperactive disorder, interpersonal strategies, oppositional defiant disorder, outcome, separation anxiety | Epidemiological and clinical data suggest that oppositional defiant disorder (ODD) is a frequent outcome of infantile attention deficit/hyperactivity disorder (ADHD), whether ADHD is primarily pure or comorbid with ODD. While the reasons for this evolution remain unknown, it is suggested that ADHD implies a specific pattern of interaction between the hyperactive child and his/her parents, based on the externalization and projection on the parent of the child’s psychological function of attention: the more the child lacks attention, anticipation and self-control, the more the parents must be attentive, anticipative and controlling on the child’s behalf. This pattern may be triggered by separation anxiety, where the child searches for parental control since it implies physical closeness and therefore is incompatible with separation. ADHD may thus serve as a strategy with the function to control parents and prevent separation. Because of its control component, the parent/child relationship is at risk to turn out into a destructive vicious circle, impulsive/disruptive behaviour reinforcing control/punishment and vice versa. This is hypothesised to be the pathway to ODD. The hypothesis is developed and discussed with reference to the case of a boy suffering from ADHD when he was 6 years old, and who was re-examined 7 years later when presenting ODD. |
| **Sourgen. (2017). La thérapie familiale psychanalytique a-t-elle sa place dans le traitement du TDAH?**  *“Does psychoanalytic family therapy have its place in the treatment of ADHD? A child with a great destiny.”*  Keywords: ADHD, fusionnal link, incestuous atmosphere, ascendancy, maniac defences, psychoanalytic family therapy. | ADHD is a behavioral disorder that appears to be linked to a genetic peculiarity leading to a metabolic disorder in the neurotransmitters. This psychophysiological approach generally implicates drug therapy associated with multiple rehabilitations. The development of a psychoanalytic family therapy (PFT) opens up another perspective. Childhood disorder can be considered a mere symptom of the family, which is illustrated in an example of a mother-child link full of incestuous atmosphere, ambivalence and reciprocal influence. PFT, through the approach to defensive mechanisms that are transferred into the interaction between the mother, her child and the therapist, appears to be one of the most suitable treatment approaches to ADHD, at the same time avoiding to conceptualize the child a sick person or depicturing his environment as a culprit. |
| **Duc Marwood. (2020). Les contes en thérapie avec des enfants souffrant d’hyperactivité et de déficit de l’attention.**  *‘’Tales in therapies with children who have been diagnosed with ADDH’’*  Keywords: Attention deficit disorder with hyperactivity, child psychology, diagnosis, therapeutic processes, treatment, childhood (birth-12 yrs) | This article discusses the use of tales in therapies with children who have been diagnosed with ADHD. It develops the means through which tales are chosen and how a therapeutic process is being developed in order to encourage the emergence of a dreaming space for the child. This therapeutic method aims to create a space where children’s thinking is facilitated, and ultimately their ability to express feelings. Finally, this article reveals how tales constitutes an inter-subjective space in which turmoil is replaced by dialogue. |
| **Metz & Thévenot. (2010). Instabilité psychomotrice des enfants : trouble ou symptôme ?**  *‘’Hyperactivity in children– disorder or symptom?’’*  Keywords: clinical psychology, hyperactive, projective tests, psychic suffering, psychotherapy. | For some years, children’s behavioural problems constitute a growing motive for consulting. The problems are eiher referred to as hyperactivity, a pathology of an ill body that needs to be treated, or as the behavioural expression of children born in an era characterized by a lack of reference points. However, the clinical approach focuses on the singularity of each subject, whose symptom constitutes the expression of unconscious conflict. The authors present two cases: Emilie from clinical research, and Bertrand, from psychotherapy; both of them presenting with behavioural problems. From research conducted on a population of children known as hyperactive, we examined the contribution of projective methods in a clinical setting. Data was collected by examining projective tests from children, and through non-structured interviews conducted with their parents. The results showed a figure of the child in today’s society: a child to control. The group of children was heterogeneous, and the particular case of Emilie highlighted the suffering and the psychological fragility of a child that specialists as well as the mother perceives as a person to rehabilitate. Furthermore, Bertrand’s case reveals that the child’s hyperactivity should be considered further in relation to family history and dynamics. This case illustrates how the symptom is of value for the child and his parents, revealing stakes that have implications for clinical work with both parts. |
| **Bourrat. (2004). Hyperexcitabilité, hyperactivité et traumatisme**  *‘’Hyperexcitement, hyperactivity and traumatism’’*  Keywords: excitement, symbolization, traumatism through generation, hyperactivity, psychotherapy (mother and baby) | As a child psychiatrist, the author met parents and their babies who consulted for sleeping difficulties and/or hyperactivity. In addition to their inability to find rest, frequent traumatic events were found in the families’ history, including perinatal deaths or antecedents of child death in the parents’ generation. The present work suggests that there might be a link between the hyperactive symptom the baby displays in consultation and the maternal psychic functioning due to trauma. While there has been much interest in maternal depression and its relation to the parents-child interaction, fewer studies have been undertaken with a specific focus on traumatic situations that the mother suffers from, and the impact these have for parenting. This lack of research misses out on relevant issues such as mental sideration (i.e., the inability to make sense of traumatic events) and how such situations forces mothers to defend themselves against cues of frightening experiences that they are unable to process. Moreover, a case study suggests that the persistent difficulties a mother met in making sense of her 6 months old baby’s motor expression resulted from the closest connection body expression had with traumatic, non-integrated experiences. As a consequence, her inability to host her baby’s movements deprived the latter of a meaningful feedback on his own presence. In a circular way, the interaction led to merely physical responses (walking with the baby in one’s arms, playing with him, or sitting him in a baby walker), which at once produced endless movements and exhaustion on both sides. As such, this interaction prevented the parents’ ability to make sense of the mother’s helplessness and the child’s unrest. The present observation supports the importance of undertaking an early mother–baby therapy that can restore the thought capacity in the parents. |
| **Guinard. (2012). Le travail du pulsionnel et ses différentes figures chez des enfants dits ”TDAH”**  ‘’*The work of instinct and its different facets in children labelled with ADHD.’’*  Keywords: agitation, inattention, excitation. | This article discusses research that aims to apprehend the treatment modalities for instinctual excitation in children diagnosed with Attention Deficit and Hyperactivity Disorder (ADHD). Since this excitation constitutes the most visible phenomenon in the clinical encounter, the author attempts to distinguish different configurations corresponding to distinct modalities of psychological treatment for the excitation. Each configuration is illustrated with the help of examples combining clinical and projective material. At the end of the study, the hypothesis proposed is that the symptomatology might be considered not only as a discharge – something often evoked concerning agitation in children – but as a compulsion, having the particularity of being generalized to the entire body. |
|  |  |
